# Factors associated with non-response and nutritional status of non-responders at 6-month post-discharge: a cohort study nested in a MUAC-based nutrition programme for acutely malnourished children in Mirriah, Niger

**DOI:** 10.3389/fpubh.2024.1357891

**Published:** 2024-08-14

**Authors:** Maguy Daures, Jérémie Hien, Cécile Cazes, Rodrigue Alitanou, Laure Saillet, Benjamin Séri, Ahmad Ag Mohamed Aly, Oumarou Maidadji, Atté Sanoussi, Aboubacar Mahamadou, Mathias Altmann, Kevin Phelan, Renaud Becquet, Susan Shepherd

**Affiliations:** ^1^National Institute for Health and Medical Research (INSERM) UMR 1219, Research Institute for Sustainable Development (IRD) EMR 271, Bordeaux Population Health Research Centre, University of Bordeaux, Bordeaux, France; ^2^The Alliance for International Medical Action (ALIMA), Niamey, Niger; ^3^PRISME-CI ANRS|MIE Research Programme, University Hospital of Treichville, Abidjan, Côte d'Ivoire; ^4^Bien Etre de la Femme et de l'Enfant (BEFEN), Niamey, Niger; ^5^Ministry of Health, Nutrition Division, Niamey, Niger; ^6^High-Commission of the Nigériens Nourrissent les Nigériens (3N) Initiative, Niamey, Niger; ^7^The Alliance for International Medical Action (ALIMA), Dakar, Senegal

**Keywords:** acute malnutrition, non response, failure to treatment, mid-upper arm circumference, Africa, Western, children

## Abstract

**Background:**

In the treatment of acute malnutrition (AM), non-response is considered a treatment failure for not meeting recovery criteria within a therapeutic window of 12–16 weeks, but this category of children is misunderstood. As current research emphasizes ways to simplify and optimize treatment protocols, non-response emerges as a new issue to enhance program efficiency.

**Methods:**

A prospective cohort study was conducted from 2019 to 2020 at two health centres in Mirriah, Niger among children aged 6–59 months with uncomplicated AM treated under the Optimising treatment for Acute MAlnutrition (OptiMA) protocol. Children who did not meet recovery criteria by 12 weeks (mid-upper arm circumference (MUAC) ≥125 mm without oedema for two consecutive weeks) were classified as non-responders. Non-responders received a home visit six-months post-discharge. Logistic regression was used to analyze factors associated with non-responders compared with children who recovered.

**Results:**

Of the 1,112 children enrolled, 909 recovered and 139 were non-responders, of which 127 (80.6%) had significant MUAC gain (mean: +9.6 mm, sd = 5.1) at discharge. Girls (adjusted hazard ratio (aHR) = 2.07, 95% CI 1.33–3.25), children <12 months of age (aHr = 4.23, 95% CI 2.02–9.67), those with a MUAC <115 mm (aHR = 11.1, 95% CI 7.23–17.4) or severe stunting (aHR = 2.5, 1.38–4.83) at admission and a negative or flat MUAC trajectory between admission and week 4 (aHR = 4.66, 95% CI 2.54–9.13) were more likely to be non-responders. The nutritional status of non-responders had generally improved 6 months after discharge, but only 40% had achieved MUAC ≥125 mm.

**Conclusion:**

Non-responders are not a homogeneous group; while most children ultimately show significant nutritional improvement, rapid hospital referral is crucial for those not gaining MUAC early in treatment. As efforts to expand MUAC-based programming progress, adapting exit criterion and/or providing additional food supplementation with smaller daily ration for children with risk factors discussed here may help improve programme efficiency without adding to the cost of treatment.

## Introduction

In 2020, acute malnutrition affected 49.5 million children aged 6 to 59 months worldwide, including 16.6% with the severest form, (1), and contributed nearly to a quarter of all annual childhood deaths (2). A quarter of this global burden is in Africa, and the largest number of children on the continent affected by acute malnutrition, 5.1 million, live in West Africa (1). The World Health Organization (WHO) defines severe acute malnutrition (SAM) as mid-upper-arm circumference (MUAC) < 115 mm or weight for height Z-score (WHZ) < −3 or the presence of nutritional oedema, and moderate acute malnutrition (MAM) as MUAC between 115 and 124 mm or WHZ between −2 and − 3 Z-score. In an effort to simplify management of acutely malnourished children, MUAC is becoming a stand-alone practical tool for all phases of nutrition programming from screening to admission, monitoring recovery, and determining discharge. Programmes using only MUAC and oedema as admission criteria have been expanding as evidence has accumulated that WHZ alone or in combination with MUAC does not offer a clear advantage over MUAC alone in identifying children at near-term risk of death (3), and that weight and MUAC gain track each other during treatment (4, 5).

Currently, malnutrition treatment protocols consider a child recovered when MUAC ≥125 mm and/or WHZ > -2 in the absence of oedema for 2 consecutive weeks is achieved (6). Projects using only MUAC and oedema as admission criteria use the same discharge criteria except for WHZ. MUAC > = 125 mm at discharge has been shown to prolong treatment duration, lead to a higher weight gain (7–9), and to reduce the risk of relapse (10, 11). While younger and smaller children (<65 cm) showed greater proportional weight gain and longer length of stay, they are the most likely not to reach the 125 mm threshold within a therapeutic window of 12–16 weeks (8). Children treated for acute malnutrition who fail to reach MUAC ≥125 mm are thus classified as « Non-responders » at programme exit (with the other outcomes being recovery, death, default or transfer).

When outpatient treatment for acute malnutrition was first introduced in the early 2000s, programmes typically did not report data on non-response. As a result, non-response is poorly described in the literature, is often wrongly perceived as treatment failure, and little is known about factors associated with non-response. Non-response rates vary according to contexts or acute malnutrition treatment protocols in place and may become more operationally important as efforts to scale-up MUAC-based programmes progress (8, 12–16). In a MUAC-based protocol in Malawi, 19% of chidlren enrolled with a MUAC <115 mm were classified as non-responders after 16 weeks of supplementation (8). In Niger, 22.7% of children admitted with MUAC<120 mm and 35.7% admitted with WHZ < -3 or MUAC <115 mm were classified as non-responders after 8 weeks of supplementation (13). The Combined Protocol for Acute MAlnutrition Study (ComPAS), a MUAC-based simplified protocol, found a non-response rate of 14.7% in the intervention arm and 13.7% in the standard arm after 16 weeks in Kenya and South Sudan (14). Meanwhile, a cohort study including 27,800 children under the ComPAS protocol in Mali reported a non-response rate of less than 1% (16).

The Optimizing treatment for acute MAlnutrition (OptiMA) strategy is a MUAC-based approach to simplify and optimize treatment of acute malnutrition in children aged 6 to 59 months. The OptiMA strategy trains mothers to use MUAC bracelets to detect acute malnutrition in their children and targets treatment to those with MUAC<125 mm or oedema using one product – ready-to-use therapeutic food (RUTF) – at a gradually reduced dose based on a child’s weight and MUAC status during treatment (16). This strategy has been successfully tested in a randomized controlled trial in the Democratic Republic of Congo (DRC) (16, 17) and in a large-scale cohort study in Burkina Faso (18). A prospective cohort study including acutely malnourished children treated under this strategy was conducted in two health zones in rural Niger in preparation for a randomised controlled trial comparing the Nigerien national nutrition protocol to two simplified protocols (ComPAS, OptiMA) (19, 20). As part of this cohort in Niger, a post-discharge follow-up visit at home was conducted for children classified as non-responders. In this article, we report a secondary analysis of children treated under the OptiMA protocol and aimed at identifying factors associated with non-response and at describing the nutritional status of children classified as non-responders 6 months after discharge from treatment.

## Methods

### Study design, setting and period

An observational cohort study of children treated with the MUAC-based OptiMA nutrition strategy was conducted in two health facilities in the Mirriah district, in south-eastern Niger. Enrolment started in July 2019 and continued until November 2019, with weekly outpatient follow-up ending in January 2020. Between April and June 2020, this cohort was extended for a subset of children classified as non-responders (defined below) at exit from treatment under OptiMA, with a home visit conducted at 6 months post-discharge.

The non-governmental organization « The Alliance for International Medical Action (ALIMA) » has supported the Ministry of Health since 2009 to provide both outpatient and inpatient medical and nutritional care to children <5 years of age in the study area. The Zinder region is particularly affected by AM, with high prevalence of SAM and MAM in 2020, at 4.9% (95CI: 4.2–5.8) and 13.0% (95 CI: 11.7–14.5), respectively (21). Mirriah is one of the most populated districts in Niger, with an estimated population of 700,000 in 2019 with 20 health zones and a district hospital located in Mirriah town.

From 2015 to 2020, ALIMA also implemented a programme to prevent the occurrence of AM in 3 of the 20 health zones, by providing monthly nutritional supplementation [20 g/day of small quantity lipid-based nutrition supplements (SQ-LNS)] to all non-malnourished children aged 6–23 months of age. The OptiMA treatment protocol was implemented in one health zone where SQ-LNS was provided and in another where it was not.

### Eligibility criteria, treatment and follow-up, and discharge criteria in OptiMA strategy

Children were considered eligible for inclusion in the OptiMA protocol if they were aged 6–59 months, had a MUAC<125 mm or bipedal oedema without medical complications, resided in either Gaffaty (no SQ-LNS provided) or Guirari (SQ-LNS provided) health zones, and had written informed consent from their legal guardian.

Children were followed at weekly consultation in health centre, where they underwent a clinical assessment including anthropometric measurements, and received a weekly RUTF ration according to the severity of their malnutrition. In contrast to the weight-based RUTF ration in the national programme, which is fixed at 150–200 kcal/kg per d (628–837 kJ/kg per day) for the course of treatment, the OptiMA RUTF ration was calibrated to the child’s degree of wasting based on the combination of MUAC status and weight. This meant that the most severely malnourished children received more nutritional support, which was gradually reduced as the child’s MUAC and weight increased. Children with MUAC <115 mm or oedema received 150 kcal/ kg per d (732 kJ/kg per day) of RUTF. Children with MUAC 115–119 mm, either at admission or during the course of treatment, received 110 kcal/kg per day (523 kJ/kg per day) of RUTF, and children with MUAC≥120 mm received 75 kcal/kg per day (314 kJ/kg per day) of RUTF (with a minimum of one sachet per day) until discharge from the OptiMA strategy. Health promotion such as counseling on recommended Infant and Young Child Feeding (IYCF) practices, family MUAC, as well as support for continued breastfeeding are also provided to mothers during the weekly outpatient visit by health workers.

All other systematic treatments, clinical care and hospitalization follow the national protocol recommendations (22). Amoxicillin 75 mg/kg/day was prescribed to all children included with MUAC <120 mm or oedema. All children received vitamin A, deworming with albendazole if older than 12 months, rapid malaria test (RDT) on enrolment and at any time during their participation if clinical signs of malaria were detected, and treated with artesunate-based combination therapy when positive.

Recovery was defined as a MUAC ≥125 mm and no oedema for two consecutive weeks, a good clinical health and a minimum stay in the programme of 4 weeks. Non-response was defined differently for children admitted with SAM or MAM, as failure to meet discharge criteria at 10 and 12 weeks, respectively. Children with MUAC <115 mm or WHZ < -3 after 10 weeks of treatment were transferred to the national standard protocol at week 11 and children with MUAC 115–124 and WHZ ≥ -3 were discharged at week 12. Non-responders were systematically visited at home in order to evaluate their nutritional status at 6 months after exit from treatment.

### Study population

This analysis includes children who either achieved recovery or were discharged as non-responders in the OptiMA protocol.

Non-responders were further divided into 2 two categories: “Treatment failure” for any child who did not gain or lost MUAC during the study period; “Slow response” for those who gained MUAC between enrolment and discharge, considering a measurement discrepancy of 2 mm (23).

### Data collection

During the weekly outpatient follow-up, socio-demographic, clinical, and anthropometric data were collected by MoH staff supervised by a project manager using the national programme’s individual outpatient record. Staff involved in data collection had been trained on the study protocol prior to enrollment. The child’s weight, MUAC, temperature, clinical symptoms and amount of RUTF ration were recorded at each weekly visit to the health centre. Length was measured at enrolment and once a month thereafter. Weight was measured to the nearest 100 g using a Salter scale, and length was measured to the nearest 0·5 cm using a height board with the child in a supine position (or standing if taller than 85 cm). MUAC was measured to the nearest mm using a MUAC bracelet demarcated in 1 mm increments. Whether a child had received SQ-LNS prior to enrolment was reported by the caregiver without specifying the duration of supplementation. Missed weekly visits were reported on the form. At each visit, supervisors ensured that the scales were correctly calibrated and that the MUAC bracelets and height boards were in good condition. All data collected were anonymised before being entered into a database created, managed and stored by the PACCI research programme in Côte d’Ivoire. Requests were sent to the study team in the field in Niger twice a month to correct any input errors: missing data, inconsistent data, aberrant data. An external monitoring visit was carried out by PACCI at both study sites in Niger.

During the home visit data collection among the non-responders, a team composed of a Community Health Worker (CHW) and a nurse supervised by the study project manager visited children at home to collect data on the nutritional status 6 months after their discharge from OptiMA protocol. A paper form was used and then entered in an anonymized database. Data on anthropometric characteristics, hospitalization and outpatient health centre visits between OptiMA programme discharge and the home visit were collected. The same tools as described above were used to collect anthropometric data at home. Children who could not been seen after two home visits attempts were considered lost to follow-up.

### Data analysis

We compared sociodemographic, nutritional and medical characteristics at enrolment and discharge between children classified as non-responders and those who recovered from the OptiMA programme. Anthropometric characteristics (MUAC, weight-for-height Z-score (WHZ), weight-for-age Z-score (WAZ) and height-for-age Z-score (HAZ)) were described at enrolment and discharge from the OptiMA programme and 6 months after discharge for children classified as non-responders. Anthropometric Z-scores were calculated using the WHO 2006 growth standards.

Continuous variables were described in terms of mean (standard deviation, sd) using independent samples t-test or Mann & Whitney test, depending on the expected conditions of use. Categorical variables were described as frequencies and compared between included and excluded children, using the Chi^2^ test or the Fisher exact test.

Logistic regression analysis to identify factors associated with non-response was performed using the RStudio “glm” package. Univariate analysis was performed by fitting a separate model for each covariate. Variables with a *p*-value ≤0.2 were included in the multivariate analysis. In addition, the association between continuous variables such as age, MUAC, WHZ, WAZ, and HAZ was examined before building the statistical model. Variables with a correlation coefficient < 0.3 were included in the model. Multivariate analysis was performed using forward stepwise regression and variables with *p*-value <0.05 were considered as statistically significant in the final model.

The “treatment failure” and “slow response” groups were compared in terms of socio-demographic, anthropometric and medical characteristics at enrolment and discharge from the OptiMA programme and 6 months after discharge for children classified as non-responders. The same tests were used as described above.

We also used mixed effects generalized additive models to fit and plot the means of the anthropometric parameters. The smooth terms in the model were represented by using penalized regression splines. Covariates included the randomization treatment, interaction terms between randomization treatment and time, adjusted on age and sex of the child at each visit. We plotted a 95% confidence interval (95% CI) band around each curve to compare the groups.

All statistical analyses were performed using the RStudio software (RStudio, Inc.).

### Ethical statement

The study was ethically approved by the Ethics Committee of Health Ministry of Niger (approval #018/2019/CNERS). Written informed consents (signature or fingerprint) were obtained from caregivers prior to enrolment for all children included in the study.

## Results

**Figure 1:**
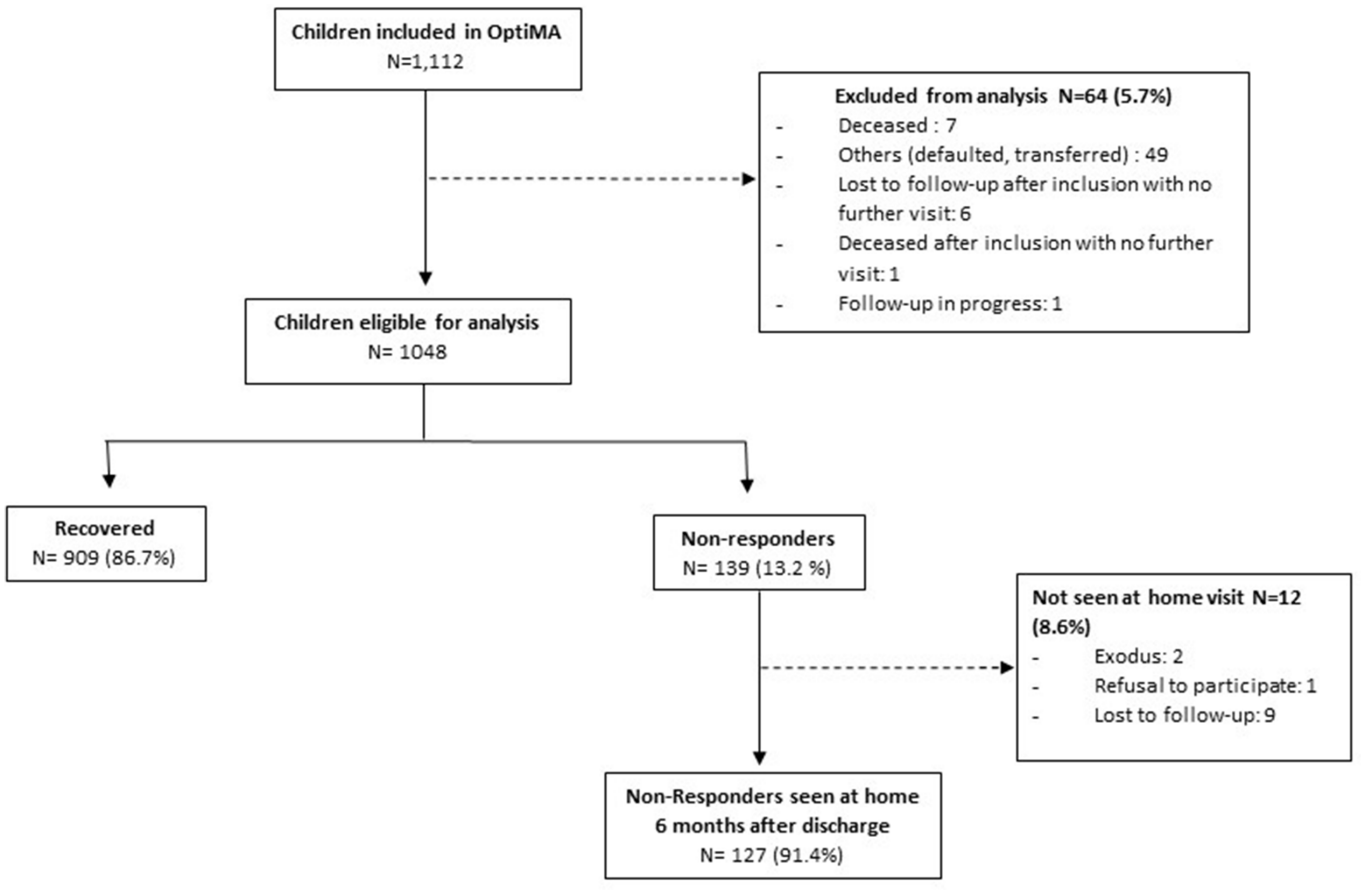
Flowchart of children included in the secondary analysis of non-response, Mirriah District, Niger.

Of the 1,112 children included in the prospective cohort study from July to November 2019, 909 (82.3%) recovered and 139 (12.6%) were classified as non-responders (Figure 1). Of the 139 children discharged as non-responders, 127 (91.4%) were seen at home 6 months after discharge (Figure 1).

Baseline characteristics of children included in this analysis, i.e., discharged as non-responders and recovered on OptiMA are shown in Table 1. Compared to recovered kids, non-responders were more likely to be girls (61.9–53.8%, *p* = 0.07) and younger (11 months IQR: 8, 15 vs 14 months IQR: 10, 20). While 83.3% of recovered children had no missed visit, this proportion was 51.1% for non-responders but, programme attendance was regular with only one missed visit for the majority of this group which is low in relation to program duration. Compared to recovered kids, non-responders were more likely to be hospitalized (51.1% vs. 8.8%, *p* < 0.001), more likely to be RDT positive during follow-up (40.3% vs. 27.3%, p < 0.001) and more likely to have either a loss or no gain in MUAC between admission and week 4 of treatment (89.9% vs. 73.0%, p < 0.001). Recovered children received a median of 52 (IQR 43, 73) sachets of RUTF during a median duration of 6 weeks (IQR 4, 9) while non-responders received a median of 119 (IQR 104, 138) sachets during a median duration of 11 weeks (IQR 9, 11). As per the study protocol, children with SAM and MAM were discharged from the programme after 10.5 weeks (sd = 1.0) and 11.3 weeks (sd = 0.5) in median to be thereafter transferred to the national protocol, respectively.

**Table 1:** Demographic, nutritional and medical characteristics of children discharged as non-responders and recovered under OptiMA protocol, Mirriah district, Niger (*n* = 1,048).

	**Overall**		**Non-responders**		**Recovered**		***p-value***
	**1,048**		***n* = 139**		***n* = 909**		
	***n***	**% or median (IQR)**	***n***	**% or median (IQR)**	***n***	**% or median (IQR)**	
**Demographic Characteristics at admission**							
Female	575	54.9	86	61.9	489	53.8	*0.07*
Age in month		14 (10, 20)		11 (8, 15)		14 (10, 20)	*< 0.001*
Age categories, months							
6–11	468	44.7	92	66.2	376	41.4	*< 0.001*
12–23	425	40.6	37	26.6	388	42.7	
≥ 24	155	14.8	10	7.2	145	16	
**Nutritional characteristics at admission**							
Breastfed	707	67.5	115	82.7	592	65.1	*< 0.001*
Received SQ-LNS prior to the index malnutrition episode	415	39.6	36	25.9	379	41.7	*< 0.001*
**Follow-up characteristics**							
Median amount of RUTF received, sachets	1,048	56 (44,86)	139	119 (104, 138)	909	52 (43,73)	*< 0.001*
Median lenght of RUTF treatment, days	1,048	6 (4,9)	139	11 (9,11)	909	6 (4,9)	*<0.001*
Number of missing visits during the follow-up							
no missing visit	828	79.0	71	51.1	757	83.3	*< 0.001*
one missing visit	149	14.2	40	28.8	109	12.0	
at least 2 missing visits	71	6.8	28	20.1	43	4.7	
SMC received at least once during the follow-up	748	71.4	110	79.1	638	70.2	*0.03*
**Clinical characteristics**							
Positive Malaria RDT at admision	154	14.7	14	10.1	140	15.4	*< 0.001*
Positive Malaria RDT during the follow-up	286	27.3	56	40.3	240	27.3	*< 0.001*
Weight loss or stagnation at least once in the first month	680	64.9	109	78.4	571	62.8	*< 0.001*
MUAC loss or stagnation at least once in the first month	789	75.3	125	89.9	664	73	*< 0.001*
Negative appetite test at least once during the follow-up	22	2.1	9	6.5	9	1.4	*< 0.001*
Fever (T > 38°c) at least once during the follow-up	169	16.1	28	20.1	141	15.5	*0.2*
**Hospitalization**							
Children hospitalized at least once	151	14.4	71	51.1	80	8.8	*< 0.001*
Main diagnosis: stagnant or loss weight	60	41.7	29	40.8	31	42.5	*0.8*
Malaria	55	36.4	25	35.2	30	37.5	*0.8*
Length of hospitalization, days	151	4 (3.0,5.0)	71	4 (3.0,5.0)	80	4 (3.0,5.0)	*0.3*

MUAC: mid-upper arm circumference. RUTF: ready-to-use therapeutic food. SQ-LNS: small quantity lipid-based nutritional supplement. RDT: rapid diagnostic test. SMC: seasonal malaria chemoprevention.

Anthropometric characteristics at baseline, discharge and 6 months post-discharge are shown in Table 2. MUAC on admission was significantly lower in non-responders than in recovered children: 110.3 mm (sd = 7.5) and 118.3 mm (sd = 4.2), respectively (*p* < 0.001). Approximately 70% of non-responders were admitted with a MUAC less than 115 mm compared to 17.6% of recovered. The mean MUAC gain between admission and discharge showed that both recovered and non-responders had a significant MUAC gain with a greater improvement for the recovered (9.1 mm, sd = 4.2) than non-responders (6.9 mm, sd = 7.7). At discharge from the OptiMA protocol, almost 60% of non-responders achieved a WHZ > = − 2. Multiple anthropometric deficits were more common in the non-responder category than in the recovered with 61.9 and 49.7% of severe stunting (*p* = 0.008) and with 88.5 and 69.6% of severe underweight (*p* < 0.001), respectively.

**Table 2:** Anthropometric caracteristics of children at admission and discharge under OptiMA protocol and 6 months post-dicharge for non-responders, Mirriah district, Niger (*n* = 1,048).

	Overall		Non-responders		Recovered		*p-value*
	1,048		*n* = 139		*n* = 909		
	*n*	% or mean (Sd)	*n*	% or mean (Sd)	*n*	% or mean (Sd)	
**MUAC characteristics at admission, discharge and 6 months post-discharge**							
MUAC at admission, mean(sd)		117.2 (5.5)		110.3 (7.5)		118.3 (4.2)	*< 0.001*
MUAC <115 mm at admission	256	24.4	98	70.5	158	17.4	*< 0.001*
Oedema (+, ++)	10	1.0	1	0.7	9	1.0	*< 0.001*
MUAC at discharged, mean (sd)		126 (4.6)		117.2 (5.6)		127.4 (2.4)	*< 0.001*
MUAC <115 mm at discharge	25	2.4	25	18.0	0	0	*< 0.001*
Average MUAC gain during OptiMA program (mm)		8.8 (4.9)		6.9 (7.7)		9.1 (4.2)	*< 0.001*
MUAC at 6 months post-discharge, mean(sd)*	–			121 (8.6)	–	–	–
MUAC <115 mm at 6 months post-discharge *	–	–	25	20.5			
MUAC > = 125 mm at 6 months post-discharge *	–	–	49	39.5	–	–	*–*
MUAC gain at 6 months after exit program*				3.6 (8.4)	–	–	*–*
***WHZ characteristics at admission. Discharge and 6 months post-discharge***							
WHZ at admission, mean (sd)		−2.6 (0.8)		−3.0 (1.0)		−2.5 (0.8)	*< 0.001*
WHZ < -3 at admission	316	30.2	71	51.1	245	27.0	*< 0.001*
WHZ at discharge, mean (sd)	–	−1.1 (0.9)		−1.7 (1.2)		−0.9 (0.8)	*< 0.001*
WHZ < -3 at discharge	23	2.2	18	12.9	5	0.6	*< 0.001*
WHZ > = − 2 at discharge	904	86.3	83	59.5	821	90.3	*< 0.001*
WHZ at 6 months post-discharge, mean(sd)*	–	–		−2 (1.1)	–	–	–
WHZ < −3 at 6 months post-discharge *	–	–	21	16.8	–	–	–
**HAZ characteristics at admission. Discharge and 6 months post-discharge**							
HAZ at admission, mean(sd)		-3 (1.3)		−3.4 (1.4)		−3.0 (1.3)	*< 0.001*
HAZ < -3 at admission	538	51.3	86	61.9	452	49.7	*< 0.008*
HAZ at discharge, mean (sd)		−3.6 (1.4)		−4.6 (1.2)		−3.5 (1.4)	*< 0.001*
HAZ < -3 at discharge	699	66.7	126	90.0	573	63.0	*< 0.001*
HAZ at 6 months post-discharge, mean(sd)*	–	–		−3.5 (1.3)	–	–	
HAZ < -3 at 6 months post-discharge *	–	–	83	66.4	–	–	
**WAZ characteristics at admission. Discharge and 6 months post-discharge**							
WAZ at admission, mean(sd)		−3.5 (0.8)		−4.1 (1.0)		−3.4 (0.8)	*< 0.001*
WAZ < -3 at admission	747	71.3	123	88.5	624	68.6	*< 0.001*
WAZ at discharge, mean (sd)		−2.8 (1.0)		−3.9 (0.8)		−2.6 (0.9)	*< 0.001*
WAZ < -3 at discharge	450	42.9	125	89.9	14	10.1	*< 0.001*
WAZ at 6 months post-discharge, mean(sd)*	-	-		−3.3 (1)	-	-	
WAZ < -3 at 6 months post-discharge *	-	-	77	61.6	-	-	

*3 children died 6 months after discharge. HAZ: height for age *Z*-score. MUAC: mid-uppe arm circumference. WAZ: weight for age *Z*-score/ WHZ: weight for height *Z*-score.

Factors associated with non-response are shown in Table 3. Girls (adjusted hazard ratio (aHR) = 2.07, 95% CI 1.33–3.25), children younger than 12 months (aHR = 4.23, 95% CI 2.02–9.67), those with a MUAC <115 mm or oedema on admission (aHR = 11.1, 95% CI 7.23–17.4) or a HAZ < -3 Z-score (aHR = 2.5, 1.38–4. 83), a negative or flat MUAC trajectory between admission and week 4 (aHR = 4.66, 95% CI 2.54–9.13) and those with a negative appetite test during follow-up (aHR = 3.42, 95% CI 1.17–9.62) were more likely to be discharged as non-responders. Children who received SQ-LNS prior to treatment with RUTF were less likely to be discharged than non-responders (aHR = 0.49, 95% CI 0.30–0.77).

**Table 3:** Factors associated with non-response of acute malnutrition treatment under OptiMA protocol, Mirriah district, Niger (*N* = 1,048).

		**Univariate analysis**			**Multivariate analysis**		
**Factors**		**HR**	**95%CI**	***p*-value**	**aHR**	**95%CI**	***p*-value**
**Demographic characrteristics**							
Sex							
	Male	1			1		
	Female	*1.39*	0.97–2.02	0.076	2.07	1.33–3.25	0.001
Age categories (months)				<0.001			<0.001
	≥ 24	1			1		
	6–11	3.55	1.88–7.44	<0.001	4.23	2.02–9.67	0.001
	12–23	1.38	0.70–3.00	0.4	1.7	0.79–3.94	0.2
							
**Anthropometric characteristics**							
MUAC categories							
	≥ 115 mm	1			1		
	< 115 mm or oedema	11.2	7.54–16.9	<0.001	11.1	7.23–17.4	<0.001
HAZ, categories				<0.001			
	≥ −2 Zscore	1			1		
	< − 3 Zscore	1.83	1.12–3.10	<0.001	2.5	1.38–4.83	0.003
	≥ − 3 and < −2 Zscore	1.21	0.68, 2.18	0.5	1.3	0.69–2.64	0.4
WHZ, categories				<0.001			
	≥ −2 Zscore	1					
	< − 3 Zscore	3.14	1.85, 5.59	<0.001	–	–	–
	≥ − 3 and < −2 Zscore	1.15	0.67, 2.08	0.6	–	–	–
WAZ, categories							
	≥ −2 Zscore	1					
	< − 3 Zscore	7.88	1.69,141.0	0.042	–	–	–
	≥ − 3 and < −2 Zscore	2.45	0.48,44.9	0.4	–	–	–
**Nutritional characteristics**							
Breastfed							
	No	1					
	Yes	2.57	1.65–4.15	<0.001	–	–	–

Received SQ-LNS							
	No	1			1		
	Yes	0.49	0.32–0.72	<0.001	0.49	0.30–0.77	0.003
**Medical characteristics**							
SMC received at least once during follow-up							
	No	1					
	Yes	1.61	1.06–2.52	0.031	–	–	–
Weight loss or stagnation weight at least once in the first month							
	No	1					
	Yes	2.15	1.42–3.34	<0.001	-	-	-
**MUAC loss or stagnation at least once in the first month**							
	No	1			1		
	Yes	3.29	1.92–6.09	<0.001	4.66	2.54–9.13	<0.001
**Negative appetite test at least once during the follow-up**							
	No				1		
	Yes	4.77	1.93–11.3	<0.001	3.42	1.17–9.62	0.021
**Fever (T > 38°c) at least once during the follow-up**							
	No	1					
	Yes	1.37	0.86–2.13	0.2	-	-	-

Number in dataframe = 1,048, Number in model = 1,048, Missing = 0, AIC = 608, C-statistic = 0.856, H&L = Chi-sq(8) 4.98 (*p* = 0.759).

Of the 139 children discharged as non-responders (Table 4), 80.6% were defined as having a slow response, with a mean MUAC gain of 9.6 mm (sd = 5.1) during the outpatient treatment period. The remaining children (19.4%) were identified as treatment failures because they had an average MUAC loss of −4.2 mm (sd = 5.1) during the treatment period. The mean MUAC at baseline was significantly lower in the children classified as slow responders than in the treatment failure group at 108.6 mm (sd = 7.0) and 117.6 mm (sd = 5.0), respectively (*p* < 0.001). Children classified as slow responders also had worse anthropometric co-deficits at enrolment, while the treatment failure group were more likely to be hospitalized (74.1–45.5%, *p* = 0.008). Factors associated with slow response were similar to those described above for non-responders (Supplementary file S1).

**Table 4:** Socio-demographic, anthropometric and medical characteristics of Non-responders according to their MUAC evolution under the OptiMA protocol, Mirriah district, Niger (*n* = 139).

	**Treatment failure**		**Slow response**		***p*-value**
	***n* = 27**		***n* = 112**		
	***n***	**% or mean (Sd) or median (IQR)**	***n***	**% or mean (Sd) or median (IQR)**	
**Demographic characteristics**					
Female	19	70.4	67	59.8	0.3
Age in month, median (IQR)		9 (8, 13)		11 (8, 16)	<0.001
Age categories (months)					
6–11	18	66.7	74	66.1	0.8
12–23	8	29.6	29	25.9	
≥ 24	1	3.7	9	8.0	
**Anthropometric characteristics at admission, discharge and 6 months post-discharge**					
***MUAC characteristics***					
MUAC at admission, mean(sd)		117.6 (5)		108.6 (7)	<0.001
MUAC <115 mm at admission	7	25.9		91	<0.001
Oedema (+, ++)	0	0	1	0.9	1
MUAC at discharge, mean(sd)		113.4 (7.3)		118.1 (4.7)	<0.001
MUAC <115 mm at discharge (mm)	10	37.0	15	13.4	0.004
Average MUAC gain during OptiMA program (mm)		- 4.2 (5.1)		9.6 (5.1)	<0.001
MUAC at 6 months post-discharge *		123.1 (8.4)		120.6 (8.6)	0.3
MUAC <115 mm at 6 months post-discharge *	4	17.4	21	21.6	0.7
MUAC > = 125 mm at 6 months post-discharge *	10	43.5	39	38.2	0.6
MUAC gain at 6 months post-discharge *		8.8 (9.2)		2.5 (7.9)	0.001
***Other anthropometric caracteristics***					
WHZ at admission, mean(sd)		−2.48 (0.7)		−3.2 (1)	<0.01
WHZ at discharge, mean(sd)		−2.6 (1.2)		−1.5 (1.2)	0.0012
WHZ at 6 months post-discharge, mean(sd)		−2.1 (0.8)		−2.0 (1.1)	0.8
HAZ, mean(sd) at admission		−2.7 (1.2)		−3.6 (1.5)	<0.01
HAZ at discharge, mean(sd)		−4.3 (1.3)		−4.7 (1.2)	0.2
HAZ at 6 months post-discharge, mean(sd)		−3.5 (1.4)		−3.5 (1.2)	0.8
WAZ, mean(sd) at admission		−3.4 (0.7)		−4.2 (0.8)	<0.001
WAZ at discharge, mean(sd)		−4.2 (0.7)		−3.8 (0.7)	0.018
WAZ at 6 months post-discharge, mean(sd)		−3.2 (0.9)		−3.3 (1)	1
**Nutritional characteristics**					
Breastfed	23	85.2	92	82.1	0.9
Received SQ-LNS	7	25.9	29	25.9	1
**Follow-up characteristics**					
Median amount of RUTF received, sachets	27	103 (93,130)	112	122 (109,139)	<0.001
Median length of RUTF treatment, weeks	27	11 (11,12)	112	10 (9,11)	<0.001
Number of missing visit during the follow-up					
No missing visit	13	48.1	58	51.8	0.4
one missing visit	6	22.2	34	30.4	
at least 2 missing visits	8	29.6	20	17.9	
SMC received at least once during the follow-up	25	92.6	85	75.9	0.06
**Clinical characteristics**					
Children hopsitalized during the follow-up	20	74.1	51	45.5	0.008
RDT + at admission	0	0	14	12.5	<0.001
RDT + during follow-up	7	25.9	49	43.8	0.09
Weight loss or stagnation weight at least once in the first month	24	88.9	85	75.9	0.1
MUAC loss or stagnation at least once in the first month	25	92.6	100	89.3	0.9
Negative appetite test at least once during the follow-up	2	7.4	7	6.2	0.7
Fever (T > 38°c) at least once during the follow-up	8	29.6	20	17.9	0.2

*3 children died during the 6 months after discharge. HAZ: height for age *Z*-score. MUAC: mid-uppe arm circumference. RUTF: ready-to-use therapeutic food. SQ-LNS: small quantity lipid-based nutritional supplement. RDT: rapid diagnostic test. SMC: seasonal malaria chemoprevention. WAZ: weight for age *Z*-score/ WHZ: Weight for height *Z*-score.

**Figure 2:**
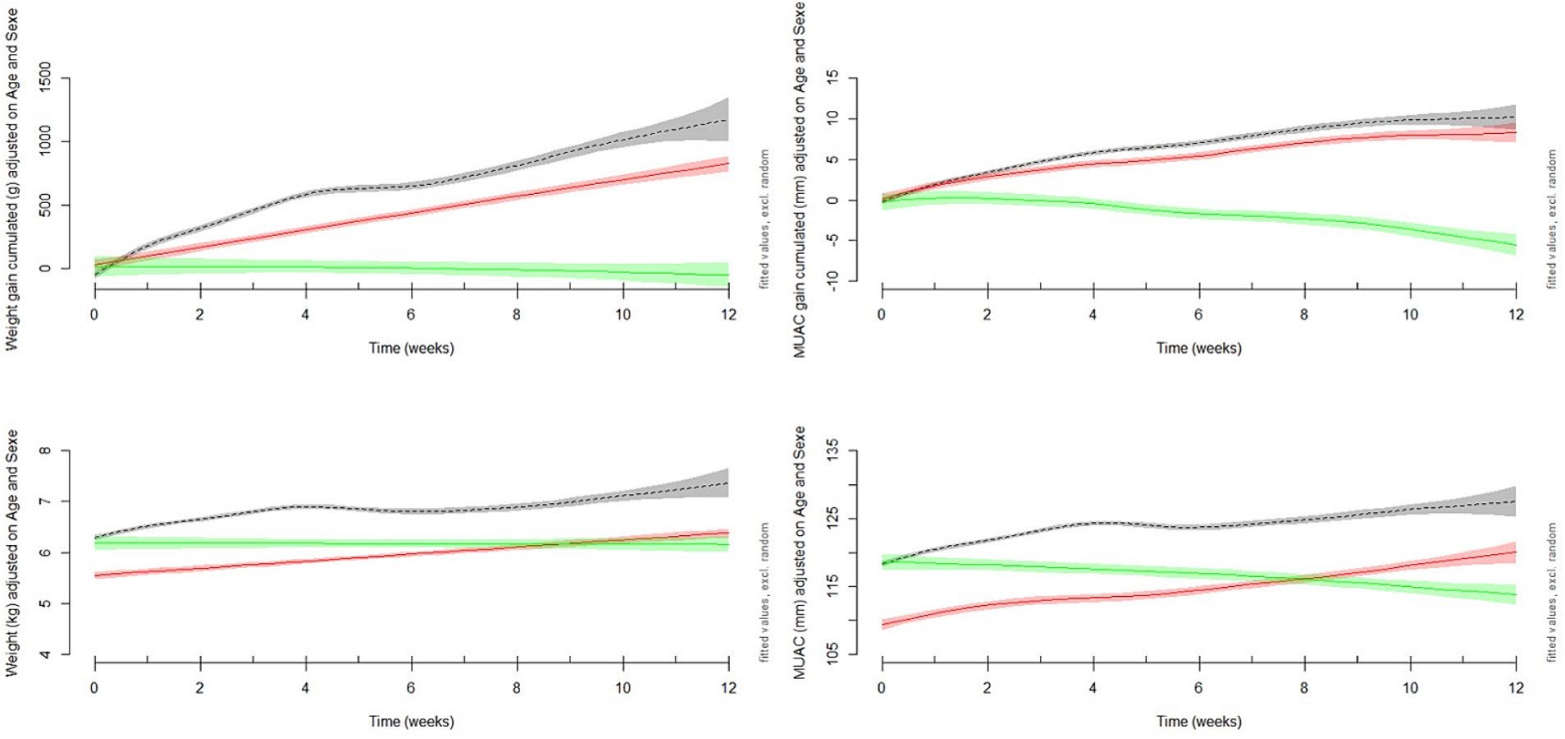
Modeled adjusted weekly means of MUAC and weight gain, and cumulative MUAC and weight gain during outpatient follow-up by recovered (*n* = 909), partial response (*n* = 112) and treatment failure groups (*n* = 27). Recovered children defined by reaching a MUAC> = 125 mm and no oedema for two consecutive weeks, a minimum of 4 weeks under treatment and no fever. The “treatment failure” includes any child who failed to gain or lost MUAC over the study period. The “slow response” includes those who gained more than 2 mm of MUAC between admission and discharge.

Growth curves adjusted for age and sex (Figure 2) showed that the trajectories of anthropometric criteria were different for the three groups from enrolment. The treatment failure group started with similar anthropometric criteria compared to the recovered children, but their weight and MUAC decreased dramatically during outpatient follow-up, whereas the “slow responders” started with lower MUAC and weight and then improved more slowly than the recovered children.

Six months after discharge from the OptiMA programme, the mean MUAC of non-responders increased from 117.2 mm (5.6) to 121.0 mm (sd = 8.5), 20.5% of them still had a MUAC<115 mm and 39.5% reached the MUAC threshold of 125 mm (Table 2). At 6 months after discharge, 66.4 and 61.6% of non-responders were still severely stunted and severely underweight, respectively. The mean MUAC gain 6 month after exit for both the slow response and treatment failure groups had generally improved, but in both groups around 60% of children still had a MUAC <125 mm 6 months after discharge from the OptiMA programme (Table 4). Three (2.4%) deaths were recorded among the non-responders including two in the treatment failure group.

## Discussion

To the best of our knowledge, this is the first description of African children who fail to meet discharge criteria at the end of acute malnutrition treatment despite regular programme attendance. In this cohort, children who failed to achieve a MUAC ≥125 mm for two consecutive weeks were divided into two distinct groups: those whose rate of MUAC gain was similar to those who recovered (9.6 mm, sd = 5.1 vs. 9.1 mm, sd = 4.2) and those whose MUAC gain was flat or negative. The vast majority (80.6%) were in first group, the slow responders. They started with a lower MUAC on admission and failed to reach the discharge threshold within the allocated treatment period. The second group, treatment failures, had a higher MUAC on admission but their anthropometric measurements declined during follow-up and they were much more likely to be hospitalized, suggesting that acute malnutrition in these cases was associated with acute and/or chronic illness. Children younger than 12 months, girls, children with a low MUAC (<115 mm) or severe stunting at enrolment, and a flat or negative MUAC trajectory between admission and week 4 of treatment were found to be at higher risk of non-response, whereas receiving SQ-LNS prior to being treated with RUTF was found as to be protective against non-response. The nutritional status of both categories of non-responders had generally improved 6 months after leaving the programme, but about 60% of these children still had a MUAC <125 mm.

This analysis provides two important findings. First, close monitoring of the response to treatment during the first few weeks of treatment is crucial. Children with a flat or negative MUAC trajectory during this period require careful evaluation for comorbidities or undiagnosed underlying illnesses (i.e., sickle cell disease, tuberculosis) (24). Second, a significant proportion of children who are monitored may not reach recovery criteria but nevertheless respond to treatment albeit with slower growth trajectories. These children likely require more sustained nutritional support to achieve and maintain healthy growth. In this light, the term ‘non-response’ in a MUAC-based programme might more usefully be redefined as acute malnutrition secondary to acute or chronic illness for the former group and ‘slow responders’ for the latter.

The ‘slow responders’ had an average WHZ at discharge of −1.5 (sd = 1.2), meaning that although they were still acutely malnourished by MUAC, they had reached the WHZ criterion for recovery. This is explained by the high prevalence of severe stunting in this group, thus these children are still considered at high risk of mortality with low weight for age (25–27) and should benefit from sustained nutritional support until MUAC is >125 mm and/or WAZ is > − 2. Routine supplementation with SQ-LNS between 6 to 23 months has a strong evidence base, including significant relative risk reductions in stunting, wasting, anaemia, and mortality, as well as improved motor skills and cognitive development (28–30). Supplementation with a smaller daily ration such as SQ-LNS (120 kcal/day) for 6 to 12 months after a minimum of 4–6 weeks of intensive treatment with RUTF, may be appropriate for these children, particularly as it was shown to protect against non-response if received prior to treatment (20).

Children at higher risk of slow response can be easily identified on admission. Multivariate analysis showed that girls under 12 months with severe stunting and a MUAC <115 mm were at highest risk. This analysis is conducted within a MUAC-based program, which has been demonstrated in several studies to be age and gender-dependent (31), resulting screening in or selection of a higher proportion for girls and younger children. When examining the WHO growth trajectory of MUAC for age, these limitations are not surprising. The threshold for girls under 18 months to be considered well-nourished (MUAC for age > −2) is below 125 mm, while for boys, the threshold exceeds 125 mm after 9 months old. Consequently, younger girls are less likely to reach the MUAC threshold at 125 mm. These findings are also consistent with previous studies describing younger and smaller children with a low MUAC at admission who have not achieved a MUAC of 125 mm within a specified timeframe (8). Children who meet these criteria and who have gained 10 mm MUAC in the acute malnutrition treatment programme, regardless of their absolute MUAC value, would be candidates for transition to a longer-term programme to provide a smaller daily ration such as SQ-LNS (120 kcal/day) over a longer period of time. This would be relatively easy to implement and would avoid the need for children to be supplemented with more expensive RUTF for long periods of time. MUAC for age could also be considered, but several studies have shown that the power of MUAC to predict mortality is independent of age, even in the youngest children (<1 year) (32, 33).

The smaller treatment failure group (19.4%) could be considered as true treatment failures, as they did not show any MUAC improvement during treatment. Those children even lost an average of 4.2 mm over the 10–12 weeks of supplementation. This was despite the fact that these children had a better nutritional status upon admission than those classified here as slow responders, with a MUAC at admission of 117 mm versus 108 mm. This group of children had more hospitalisations (74.1%) than slow responders (45.5%), suggesting that the treatment failure group may have been affected by more illness episodes during their treatment. Comorbidities were not recorded in this study but our hypothesis is consistent with a recent study showing that episodes of acute respiratory infection, diarrhoea and malaria were associated with non-response (34). Further clinical investigations such as tuberculosis or sickle cell disease should also be considered.

Six months after programme exit, the nutritional status of non-responders, both “slow responders” and “treatment failures,” had generally improved, but only 40% achieved a MUAC >125 mm 6 months after exit from treatment under the OptiMA protocol. Niger routinely records some of the worst demographic, economic and health indicators in the world, with high levels of food insecurity (35), which may partly explain the weak improvement in nutritional status 6 months after stopping treatment. Consequently, this finding supports the transition of the ‘slow responders’ to a longer-term nutrition programme.

This study is limited by the lack of documentation of case management after discharge from the OptiMA programme, so it is uncertain whether non-responders received additional RUTF between discharge and community follow-up 6 months later. Children who are admitted to the standard protocol after being discharged from the OptiMA protocol may have better nutritional status than those who are not. Whether a child had received SQ-LNS prior to the index acute malnutrition episode was simply reported by the caregiver without specifying how much was received or for how long, so we were unable to explore any association between the amount of SQ-LNS received prior to treatment and the protection against non-response. Further studies should investigate this association. There is a need for more powerful analysis using growth trajectories to confirm the group of children defined as ‘slow responders’ and ‘treatment failures’ in this study, and to evaluate a MUAC gain threshold that could be used to transfer children into a long-term supplementation programme as proposed here.

## Conclusion

Refining the definition of the ‘non-responder’ category is likely to become more operationally important as efforts to scale up MUAC-based programmes continue. Our analysis re-emphasises the importance of rapid hospital referral for children who do not gain MUAC in the first few weeks of treatment and who require intensive medical care. However, the vast majority of children who do not meet recovery criteria despite programme attendance do improve their nutritional status significantly, but with a growth trajectory that precludes them from achieving recovery within a reasonable timeframe of therapeutic feeding. A MUAC recovery threshold of 125 mm is unlikely to be realistic for stunted young girls with low MUAC. An alternative criterion, such as raw MUAC gain, could be a relevant and operational criterion for transitioning these children at highest risk of mortality to a longer-term feeding programme. The poor nutritional status 6 months after discharge also reinforces the need to provide nutritional support at lower levels and for longer periods of time in such a context as Niger.

## Data availability statement

The raw data supporting the conclusions of this article will be made available by the authors, without undue reservation.

## Ethics statement

The studies involving humans were approved by the National Ethics Committee for Health Research in Niger (Number 018/2019/CNERS July 11, 2019). The studies were conducted in accordance with the local legislation and institutional requirements. Written informed consent for participation in this study was provided by the participants’ legal guardians/next of kin.

## Author contributions

MD: Conceptualization, Formal analysis, Methodology, Writing – original draft, Writing – review & editing, Validation. JH: Conceptualization, Data curation, Formal analysis, Methodology, Supervision, Writing – original draft, Writing – review & editing, Validation. CC: Writing – review & editing. RA: Supervision, Writing – review & editing, Data collection. LS: Methodology, Supervision, Writing – review & editing. BS: Software, Writing – review & editing. AA: Resources, Writing – review & editing, Supervision. OM: Resources, Writing – review & editing, Project administration, Supervision. AS: Resources, Writing – review & editing, Supervision. AM: Writing – review & editing, Supervision. MA: Validation, Writing – review & editing, Methodology. KP: Funding acquisition, Validation, Writing – review & editing, Conceptualization. RB: Validation, Writing – review & editing. SS: Funding acquisition, Validation, Writing – review & editing, Conceptualization.

## References

[1] Levels and trends in child malnutrition: UNICEF/WHO/The World Bank Group joint child malnutrition estimates: key findings of the 2021 edition. Available at: https://www.who.int/publications-detail-redirect/9789240025257 (Accessed December 9, 2021).

[2] Black RE Victora CG Walker SP Bhutta ZA Christian P de Onis M. Maternal and child undernutrition and overweight in low-income and middle-income countries. Lancet Lond Engl. (2013) 382:427–51. doi: 10.1016/S0140-6736(13)60937-X 23746772

[3] Briend A Maire B Fontaine O Garenne M. Mid-upper arm circumference and weight-for-height to identify high-risk malnourished under-five children. Matern Child Nutr. (2012) 8:130–3. doi: 10.1111/j.1740-8709.2011.00340.x, PMID: 21951349 PMC6860828

[4] Binns P Dale N Hoq M Banda C Myatt M. Relationship between mid upper arm circumference and weight changes in children aged 6-59 months. Arch Public Health Arch Belg Sante Publique. (2015) 73:54. doi: 10.1186/s13690-015-0103-y, PMID: 26693279 PMC4685635

[5] Burza S Mahajan R Marino E Sunyoto T Shandilya C Tabrez M. Community-based management of severe acute malnutrition in India: new evidence from Bihar. Am J Clin Nutr. (2015) 101:847–59. doi: 10.3945/ajcn.114.093294, PMID: 25833981 PMC4381773

[6] WHO - Library Cataloguing-in-Publication Data. Guideline: updates on the management of severe acute malnutrition in infants and children. 1. Malnutrition. 2. Infant nutrition disorders. 3. Child nutrition disorders. 4. Guideline. I. World Health Organization. 24649519

[7] Dale NM Myatt M Prudhon C Briend A. Using mid-upper arm circumference to end treatment of severe acute malnutrition leads to higher weight gains in the most malnourished children. PLoS One. (2013) 8:e55404. doi: 10.1371/journal.pone.0055404, PMID: 23418442 PMC3572091

[8] Binns PJ Dale NM Banda T Banda C Shaba B Myatt M. Safety and practicability of using mid-upper arm circumference as a discharge criterion in community based management of severe acute malnutrition in children aged 6 to 59 months programmes. Arch Public Health Arch Belg Sante Publique. (2016) 74:24. doi: 10.1186/s13690-016-0136-x, PMID: 27307989 PMC4908708

[9] Isanaka S Hanson KE Frison S Andersen CT Cohuet S Grais RF. MUAC as the sole discharge criterion from community-based management of severe acute malnutrition in Burkina Faso. Matern Child Nutr. (2019) 15:e12688. doi: 10.1111/mcn.12688 30194814 PMC6585742

[10] Somassè YE Dramaix M Bahwere P Donnen P. Relapses from acute malnutrition and related factors in a community-based management programme in Burkina Faso. Matern Child Nutr. (2016) 12:908–17. doi: 10.1111/mcn.12197, PMID: 26059267 PMC6860074

[11] Stobaugh HC Mayberry A McGrath M Bahwere P Zagre NM Manary MJ. Relapse after severe acute malnutrition: a systematic literature review and secondary data analysis. Matern Child Nutr. (2019) 15:e12702. doi: 10.1111/mcn.12702, PMID: 30246929 PMC6587999

[12] Linneman Z Matilsky D Ndekha M Manary MJ Maleta K Manary MJ. A large-scale operational study of home-based therapy with ready-to-use therapeutic food in childhood malnutrition in Malawi. Matern Child Nutr. (2007) 3:206–15. doi: 10.1111/j.1740-8709.2007.00095.x, PMID: 17539889 PMC6860523

[13] Aguayo VM Badgaiyan N Qadir SS Bugti AN Alam MM Nishtar N. Community management of acute malnutrition (CMAM) programme in Pakistan effectively treats children with uncomplicated severe wasting. Matern Child Nutr. (2018) 14. doi: 10.1111/mcn.12623 PMC6866122 30499254

[14] Garba S Salou H Nackers F Ayouba A Escruela M Guindo O. A feasibility study using mid-upper arm circumference as the sole anthropometric criterion for admission and discharge in the outpatient treatment for severe acute malnutrition. BMC Nutr. (2021) 7:47. doi: 10.1186/s40795-021-00448-w, PMID: 34380573 PMC8359601

[15] Bailey J Opondo C Lelijveld N Marron B Onyo P Musyoki EN. A simplified, combined protocol versus standard treatment for acute malnutrition in children 6-59 months (ComPAS trial): a cluster-randomized controlled non-inferiority trial in Kenya and South Sudan. PLoS Med. (2020) 17:e1003192. doi: 10.1371/journal.pmed.1003192, PMID: 32645109 PMC7347103

[16] Kangas ST Marron B Tausanovitch Z Radin E Andrianarisoa J Dembele S. Effectiveness of acute malnutrition treatment at health center and community levels with a simplified, combined protocol in Mali: an observational cohort study. Nutrients. (2022) 14:4923. doi: 10.3390/nu14224923, PMID: 36432609 PMC9699530

[17] Cazes C Phelan K Hubert V Boubacar H Bozama LI Sakubu GT. Simplifying and optimising the management of uncomplicated acute malnutrition in children aged 6-59 months in the Democratic Republic of the Congo (OptiMA-DRC): a non-inferiority, randomised controlled trial. Lancet Glob Health. (2022) 10:e510–20. doi: 10.1016/S2214-109X(22)00041-9, PMID: 35303461

[18] Cazes C Phelan K Hubert V Boubacar H Bozama LI Sakubu GT. Optimising the dosage of ready-to-use therapeutic food in children with uncomplicated severe acute malnutrition in the Democratic Republic of the Congo: a non-inferiority, randomised controlled trial. EClinicalMedicine. (2023) 58:101878. doi: 10.1016/j.eclinm.2023.101878, PMID: 36915287 PMC10006445

[19] Daures M Phelan K Issoufou M Kouanda S Sawadogo O Issaley K. New approach to simplifying and optimising acute malnutrition treatment in children aged 6-59 months: the OptiMA single-arm proof-of-concept trial in Burkina Faso. Br J Nutr. (2020) 123:756–67. doi: 10.1017/S0007114519003258, PMID: 31818335 PMC7054246

[20] Daures M Hien J Phelan K Boubacar H Atté S Aboubacar M. Simplifying and optimising management of acute malnutrition in children aged 6 to 59 months: study protocol for a 3 arms community-based individually randomised controlled trial in decentralised Niger. Trials. (2022) 23:89. doi: 10.1186/s13063-021-05955-6, PMID: 35090531 PMC8796195

[21] Phelan K Seri B Daures M Yao C Alitanou R Aly AAM. Treatment outcomes and associated factors for hospitalization of children treated for acute malnutrition under the OptiMA simplified protocol: a prospective observational cohort in rural Niger. Front Public Health. (2023) 11:1199036. doi: 10.3389/fpubh.2023.1199036, PMID: 37475774 PMC10354363

[22] INS Niger. National nutrition survey using the SMART 2020 methodology. Available at: https://www.stat-niger.org/wp-content/uploads/nutrition/RAPPORT_SMART_Niger_2020_VF.pdf (Accessed December 9, 2021).

[23] Ministry of Public Heath. National protocol of acute malnutrition treatment. Available at: https://fr.scribd.com/document/463099928/Protocole-nat-PEC-Nutrition-Niger-2016-VF (Accessed January 16, 2023).

[24] Rana R Barthorp H McGrath M Kerac M Myatt M. Mid-upper arm circumference Tapes and measurement discrepancies: time to standardize product specifications and reporting. Glob Health Sci Pract. (2021) 9:1011–4. doi: 10.9745/GHSP-D-21-00273, PMID: 34933994 PMC8691892

[25] Jones KDJ Berkley JA. Severe acute malnutrition and infection. Paediatr Int Child Health. (2014) 34:S1–S29. doi: 10.1179/2046904714Z.000000000218 25475887 PMC4266374

[26] McDonald CM Olofin I Flaxman S Fawzi WW Spiegelman D Caulfield LE. The effect of multiple anthropometric deficits on child mortality: meta-analysis of individual data in 10 prospective studies from developing countries. Am J Clin Nutr. (2013) 97:896–901. doi: 10.3945/ajcn.112.047639 23426036

[27] Wright CM Macpherson J Bland R Ashorn P Zaman S Ho FK. Wasting and stunting in infants and young children as risk factors for subsequent stunting or mortality: longitudinal analysis of data from Malawi, South Africa, and Pakistan. J Nutr. (2021) 151:2022–8. doi: 10.1093/jn/nxab054, PMID: 33830247 PMC8245889

[28] Stewart CP Wessells KR Arnold CD Huybregts L Ashorn P Becquey E. Lipid-based nutrient supplements and all-cause mortality in children 6-24 months of age: a meta-analysis of randomized controlled trials. Am J Clin Nutr. (2020) 111:207–18. doi: 10.1093/ajcn/nqz262, PMID: 31697329

[29] Dewey KG Stewart CP Wessells KR Prado EL Arnold CD. Small-quantity lipid-based nutrient supplements for the prevention of child malnutrition and promotion of healthy development: overview of individual participant data meta-analysis and programmatic implications. Am J Clin Nutr. (2021) 114:3S–14S. doi: 10.1093/ajcn/nqab279, PMID: 34590696 PMC8560310

[30] Prado EL Arnold CD Wessells KR Stewart CP Abbeddou S Adu-Afarwuah S. Small-quantity lipid-based nutrient supplements for children age 6-24 months: a systematic review and individual participant data meta-analysis of effects on developmental outcomes and effect modifiers. Am J Clin Nutr. (2021) 114:43S–67S. doi: 10.1093/ajcn/nqab277, PMID: 34590116 PMC8560311

[31] Hall G Chowdhury S Bloem M. Use of mid-upper-arm circumference Z scores in nutritional assessment. Lancet Lond Engl. (1993) 341:1481. doi: 10.1016/0140-6736(93)90927-9 8099180

[32] Berkley J Mwangi I Griffiths K Ahmed I Mithwani S English M. Assessment of severe malnutrition among hospitalized children in rural Kenya: comparison of weight for height and mid upper arm circumference. JAMA. (2005) 294:591–7. doi: 10.1001/jama.294.5.591, PMID: 16077053

[33] Myatt M Khara T Collins S. A review of methods to detect cases of severely malnourished children in the community for their admission into community-based therapeutic care programs. Food Nutr Bull. (2006) 27:S7–S23. doi: 10.1177/15648265060273S302, PMID: 17076211

[34] Kangas ST Salpéteur C Nikièma V Ritz C Friis H Briend A. Predictors of time to recovery and non-response during outpatient treatment of severe acute malnutrition. PLoS One. (2022) 17:e0267538. doi: 10.1371/journal.pone.0267538, PMID: 35639683 PMC9154090

[35] Garenne M. Tendances de la mortalité et de l’état nutritionnel au Niger. Juin 2021. FERDI WP n°294.

