# Supplementary material for: Factors associated with non-response and nutritional status of non-responders at 6-month post-discharge: a cohort study nested in a MUAC-based nutrition programme for acutely malnourished children in Mirriah, Niger
Source: Front Public Health. 2024 Aug 14;12:1357891. doi: 10.3389/fpubh.2024.1357891 (PMC11349736; doi:10.3389/fpubh.2024.1357891)
Supplement: Supplementary file 1 [file Table_1.DOCX]

**Supplementary file 1**: Factors associated with slow-response of acute malnutrition treatment under OptiMA protocol, Mirriah district, Niger (N=1,021)

|  | | **Multivariate analysis** | | |
| --- | --- | --- | --- | --- |
| **Factors** |  | **aHR** | **95%CI** | **p-value** |
| **Demographic characrteristics** | |  |  |  |
|  |  |  |  |  |
| **Sex** | |  |  |  |
|  | Male | 1 |  |  |
|  | Female | 2,03 | 1.23- 3.40 | 0,006 |
|  |  |  |  |  |
| **Age categories (months)** | |  |  | <0.001 |
|  | ≥ 24 | 1 |  |  |
|  | 6-11 | 4,8 | 2.14- 11.8 | <0.001 |
|  | 12-23 | 1,72 | 0.74-4.38 | 0,2 |
|  |  |  |  |  |
| **Anthropometric characteristics** | |  |  |  |
| **MUAC categories** | |  |  |  |
|  | > 115 mm | 1 |  |  |
|  | < 115 mm or oedema | 20,1 | 11.9-35.6 | <0.001 |
|  |  |  |  |  |
| **HAZ, categories** | |  |  |  |
|  | ≥ -2 Zscore | 1 |  |  |
|  | < - 3 Zscore | 3,54 | 1.70-7.81 | 0,001 |
|  | ≥- 3 and <-2 Zscore | 1,46 | 0.64-3.40 | 0,4 |
| **WHZ, categories** | |  |  |  |
|  | ≥ -2 Zscore |  |  |  |
|  | < - 3 Zscore | - | - | - |
|  | ≥- 3 and <-2 Zscore | - | - | - |
| **WAZ, categories** | |  |  |  |
|  | ≥ -2 Zscore |  |  |  |
|  | < - 3 Zscore | - | - | - |
|  | ≥- 3 and <-2 Zscore | - | - | - |
| **Nutritional characteristics** | |  |  |  |
| **Breastfed** |  |  |  |  |
|  | No |  |  |  |
|  | Yes | - | - | - |
|  |  |  |  |  |
| **Received SQ-LNS** | |  |  |  |
|  | No | 1 |  |  |
|  | Yes | 0,48 | 0.28-0.81 | 0,007 |
|  |  |  |  |  |
| **Medical characteristics** | |  |  |  |
| **SMC received at least once during the follow-up** | |  |  |  |
|  | No |  |  |  |
|  | Yes | - | - | - |
|  |  |  |  |  |
| **Weight loss or stagnation weight at least once in the first month** | |  |  |  |
|  | No |  |  |  |
|  | Yes | - | - | - |
|  |  |  |  |  |
| **MUAC loss or stagnation at least once in the first month** | |  |  |  |
|  | No | 1 |  |  |
|  | Yes | 4,56 | 2.35-9.55 | <0.001 |
|  |  |  |  |  |
| **Negative appetite test at least once during the follow-up** | |  |  |  |
|  | No | 1 |  |  |
|  | Yes | 4,15 | 1.14-14.3 | 0,027 |
|  |  |  |  |  |
| **Fever (T>38°c) at least once during the follow-up** | |  |  |  |
|  | No |  |  |  |
|  | Yes | - | - | - |

Number in dataframe = 1,016, Number in model = 1016, Missing = 0, AIC = 473.7, C-statistic = 0.899, H&L = Chi-sq(8) 4.97 (p=0.761)
